# Understanding the Mechanisms of Positive Microbial Interactions That Benefit Lactic Acid Bacteria Co-cultures

**DOI:** 10.3389/fmicb.2020.02088

**Published:** 2020-09-04

**Authors:** Fanny Canon, Thibault Nidelet, Eric Guédon, Anne Thierry, Valérie Gagnaire

**Affiliations:** ^1^STLO, INRAE, Institut Agro, Rennes, France; ^2^SPO, INRAE, Montpellier SupAgro, Université de Montpellier, Montpellier, France

**Keywords:** positive interactions, co-culture, metabolic dependencies, lactic acid bacteria, cross-feeding, public goods, microbial community

## Abstract

Microorganisms grow in concert, both in natural communities and in artificial or synthetic co-cultures. Positive interactions between associated microbes are paramount to achieve improved substrate conversion and process performance in biotransformation and fermented food production. The mechanisms underlying such positive interactions have been the focus of numerous studies in recent decades and are now starting to be well characterized. Lactic acid bacteria (LAB) contribute to the final organoleptic, nutritional, and health properties of fermented food products. However, interactions in LAB co-cultures have been little studied, apart from the well-characterized LAB co-culture used for yogurt manufacture. LAB are, however, multifunctional microorganisms that display considerable potential to create positive interactions between them. This review describes why LAB co-cultures are of such interest, particularly in foods, and how their extensive nutritional requirements can be used to favor positive interactions. In that respect, our review highlights the benefits of co-cultures in different areas of application, details the mechanisms underlying positive interactions and aims to show how mechanisms based on nutritional interactions can be exploited to create efficient LAB co-cultures.

## Introduction

It is widely acknowledged that microorganisms have colonized most natural ecosystems and that no single strain grows in isolation, so that microbes are intertwined and constantly interacting. Scientists are now trying to understand the mechanisms underlying these interactions in order to better control microbial communities and exploit them in a wide variety of bioprocesses.

**FIGURE 1:**
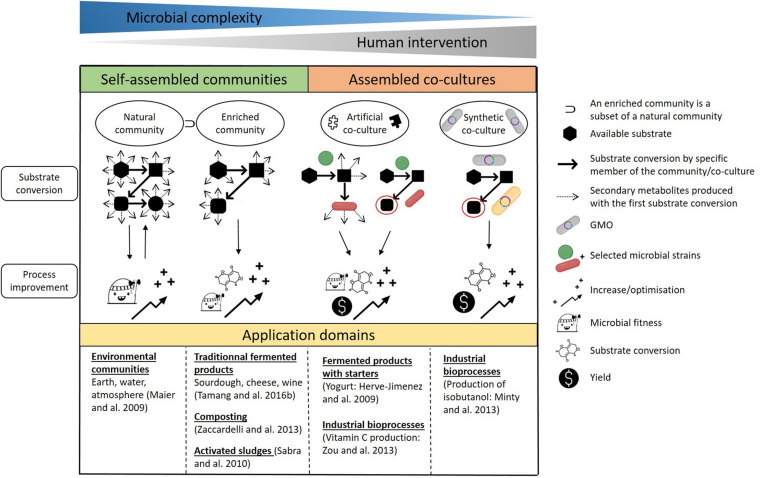
Types of microbial communities and co-cultures, their use, benefits and some examples of their areas of application. Natural community: a self-assembled association of environmental microbes found in various ecosystems. Enriched natural community: a selection of specific microbes belonging to a natural community. Artificial co-culture: an association of microorganisms that may not necessarily be found in nature. Synthetic co-culture: an association of microorganisms in which at least one of the strains is a genetically modified organism.

Before detailing how microorganisms interact together, it is crucial to define the numerous terms used in the literature which refer to their association, such as microbial community or consortium, and mixed or co-cultures. As illustrated in Figure 1, we have chosen to use the term natural community when referring to self-assembled communities of environmental microbes in various ecosystems (Rodríguez Amor and Dal Bello, 2019), and co-culture when referring to man-made associations of microorganisms (Zhang and Wang, 2016). We also use ‘assembly’ as an umbrella term which encompasses both self-assembled communities and assembled co-cultures. Biotechnological processes are reliant on three types of assemblies: enriched natural communities, artificial co-cultures and synthetic co-cultures (Figure 1). Here, artificial co-cultures refer to cultures composed of microorganisms that are generally not found together in nature, whereas synthetic co-cultures concern associations of microorganisms in which at least one of the strains is a genetically modified organism (GMO). Depending on the type of microbial assembly, the aim is (1) to increase or decrease concentrations in the targeted molecules, described as overyielding (Rapaport et al., 2020), by using the division of labor (DOL), or/and (2) to multiply the functions expressed compared to monocultures. Regardless of the objectives, in each case the outcomes are reliant on positive interactions between the microorganisms that also enhance their fitness. For this reason, negative interactions, e.g., competition, cheating or parasitism, will not be covered in this review.

Major efforts have been made to understand the mechanisms affecting the association of microorganisms in order to improve process outputs. The need for direct contact (Zengler and Palsson, 2012), use of quorum sensing (QS) (Rul and Monnet, 2015), environmental adjustments (Cheirsilp et al., 2003), the sharing of public goods (Cavaliere et al., 2017) and cross-feeding (D’Souza et al., 2018) have all been described during recent decades as possible ways to drive interactions among microbial communities.

The value of co-cultivation has been exploited in many fields, from wastewater treatments based on enriched microbial communities (Cydzik-Kwiatkowska and Zielińska, 2016) to the production of molecules of interest such as vitamin C using artificial or/and synthetic co-cultures (Zou et al., 2013; Wang et al., 2016) or a huge number of fermented foods produced worldwide. Increasing microorganism fitness, product functionalities and the production of specific molecules is crucial in the area of food applications. For example, it has been shown that co-cultures can enhance levels of peptides and amino acids (Gobbetti et al., 1994), organic acids (Settachaimongkon et al., 2014) and volatile compounds (Álvarez-Martín et al., 2008) in fermented foods, and contribute to more rapid microbial growth when compared to monocultures (Stadie et al., 2013).

Lactic acid bacteria (LAB) are ubiquitous in food fermentation and they have positive effects on the organoleptic, health, nutritional and sanitary properties of products. However, co-cultures of LAB seem to have been somewhat neglected because studies on their interactions are scarce in the literature. As reviewed by García et al. (2019), LAB are often found to be self-assembled with yeasts in fermented foods, or to a lesser extent with propionic acid bacteria (PAB) (e.g., in cheeses) or with acetic acid bacteria (e.g., in kombucha). Consequently, LAB-yeast interactions are the most widely described in the literature, such as in sourdough (Gobbetti, 1998) or kefir (Stadie et al., 2013), and have also been studied in chemically defined media (CDM) (Ponomarova et al., 2017; Carbonetto et al., 2020). The exception is the co-culture of *Lactobacillus delbrueckii* subsp. *bulgaricus* and *Streptococcus thermophilus* added in yogurt, which is well characterized (Sieuwerts et al., 2008) and offers a perfect example which asserts that LAB co-cultures are of value.

The aim of this review is to highlight the optimum ways available at present that could be used to create positive interactions between LAB. We start with an overview of the different types of positive interactions in various existing microbial assemblies. We then address the added value of co-cultivation and explain which mechanisms govern the positive interactions encountered in microbial communities and co-cultures. Thirdly, we focus specifically on the possible strategies that could be used to assemble LAB. And finally, we highlight the particular role of nutritional interactions in LAB insofar as we consider their nutritional requirements to be the best lever to create positive interactions and new LAB co-cultures.

## Positive Interactions in Co-cultures: Potential Usefulness, Types and Mechanisms

### Microbial Communities and Co-cultures: Purposes and Benefits

As the saying goes, there is strength in numbers, whether this concerns microbial communities or man-made co-cultures. It is clear that natural transformations, for example the mineralization of organic matter in soils, can occur because microorganisms are able to grow in concert. Moreover, as stated by Ghosh et al. (2016), “*the application of co-culture in different bioprocesses can be more advantageous than monocultures from the perspective of broader array of substrate utilization, coupled metabolic performance and higher combined yield*”. Thus, co-cultivation can be used for two purposes: to enable substrate conversion and improve process performance (Figure 1).

We can distinguish two types of microbial assembly: self-assembled communities and assembled co-cultures. The first gathers natural communities, enriched or not, which are spontaneous associations of microorganisms within a specific biotope. They present the highest complexity in terms of the microbial species present and hence of the resulting interactions. The dynamics of natural communities are governed by natural selection. Evolution occurs in such a way that there is a constant back and forth between the converted substrates available and the improvement in fitness of established microorganisms. Microbial interactions play a key part in improving the fitness of members in the population.

Natural microbial communities are exploited in numerous bioprocesses. It is possible to drive the conversion process by selecting certain microbial species under specific conditions. The change in microbial diversity implies that there are qualitatively fewer opportunities for substrate conversion and fewer secondary metabolites are produced overall when compared to natural communities. Enriched natural communities are oriented toward targeted results, meaning that only a few of the transformations possible are favored. This is the case in traditional fermented foods such as cheese or kimchi, as well as in composting or waste treatment. For example, methane can be produced from sludge under anaerobic conditions. The transformation of organic waste into biogas is considered to occur in four stages. During the first stage (hydrolysis), biological macromolecules are broken down into oligo- or monomers that are transformed during the second stage (acidogenesis) into volatile organic acids, alcohols, aldehydes, ketones, CO_2_ and H_2_. During the third stage (acetogenesis), the molecules produced in stage 2 are metabolized into acetic acid as well as some CO_2_ and H_2_. And during the last stage (methanogenesis), CH_4_ is formed via the decarboxylation of acetate and the methanization of CO_2_ and H_2_ (Sabra et al., 2010).

The second type of microbial assembly gathers both types of artificial and synthetic co-cultures, which require human intervention and display less microbial complexity than natural communities. Jagmann and Philipp (2014) stated that “*it is a feasible approach in biotechnology to compose microbial communities* (here referred to as co-cultures) *that either consist of wild type strains that would not necessarily co-exist in nature or of one or more genetically engineered strains.*” Microorganisms are associated in order to reduce their metabolic burden by creating a division of labor. The energy-costly pathways that require cellular building blocks and ATP are divided between multiple strains (Roell et al., 2019; Wang et al., 2020).

Artificial co-cultures are assembled for two purposes. The first is to exploit the functions expressed by each strain. This is of particular interest when developing fermented food products in which the combined activity of microbial co-cultures is responsible for their characteristic flavor and texture (Garrigues et al., 2013; Bachmann et al., 2015). For example, the mutualistic behavior of *S. thermophilus* and *L. delbrueckii subsp. bulgaricus* during the manufacture of yogurt results in improved quality and stability of the final product when compared to monocultures (Herve-Jimenez et al., 2009). The second purpose for artificial co-culture assembly is to seek the production of a specific end-product, an approach that is relevant in fields such as the production of bio-energies and bio-chemicals (Ghosh et al., 2016). One example is the production of vitamin C, where *Ketogulonicigenium vulgare* converts L-sorbose into 2-keto-L-gulonic acid (2-KLG), the precursor of vitamin C, while *Bacillus megaterium* supplies growth factors to enable the growth of *K. vulgare* and the production of 2-KLG (Zou et al., 2013). In both cases, positive interactions between microorganisms are paramount to ensure optimized substrate conversion, production of the secondary metabolites anticipated, a higher yield and/or improved microbial fitness.

Synthetic co-cultures are defined as an association of at least one GMO with other microorganisms or several GMOs. Their application is recent and mostly concerns the production of single end-products. Synthetic co-cultures are designed to increase yields and optimize substrate conversion. These goals are reliant on the positive interactions that occur between the microorganisms, which are ensured by creating metabolic dependency between them. In recent years, such metabolic dependencies have been shown to be one of the most promising ways to produce bio-energies. For example, to when producing isobutanol from cellulosic biomass, the biological functions necessary are divided between two specialists: a fungal cellulolytic specialist, *Trichoderma reesei*, which secretes cellulase enzymes to hydrolyze lignocellulosic biomass into soluble saccharides, and a fermentation specialist, a GMO *Escherichia coli* strain which metabolizes soluble saccharides into the desired product (Minty et al., 2013). It is important to mention that this use of GMOs is not conceivable for food applications, especially in Europe.

One fundamental difference between food applications and other bioprocesses is worth mentioning. In fermented food production, the composition of the initial medium is relatively simple, and the objective is to attain a complex balance between a large number of molecules such as organic acids, volatile and bioactive compounds, leading to improved shelf life, sensory qualities and health benefits of the food products. Other biotechnological processes tend to do the opposite: the initial medium is complex and the aim is to simplify this by producing a single end-product such as methane.

### Positive Interactions That Occur in the Microbial World

**FIGURE 2:**
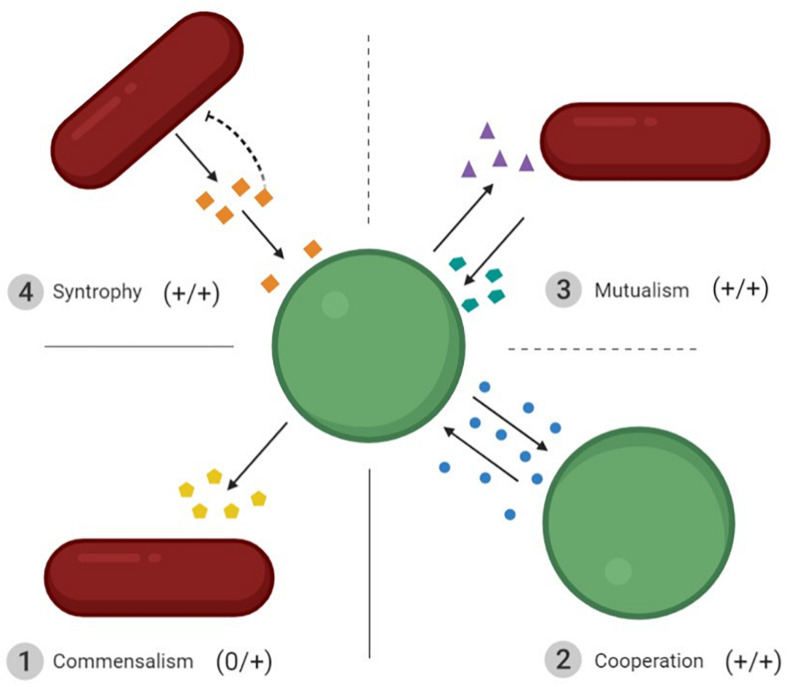
Schematic representation of the four types of interactions resulting in positive outcomes for the microorganisms involved. ➀ Commensalism: increased fitness of one interacting partner without affecting the second. ➁ Cooperation: the two interacting partners share the same phenotype and improve each other’s fitness. ➂ Mutualism: the increased fitness of two interacting partners that do not benefit from the same molecules. ➃ Syntrophy: one cell benefits from the metabolites produced by the other, and meanwhile removes the inhibition induced by these metabolites for the producer. The dotted lines between mutualism and syntrophy, and mutualism and cooperation, mean that they are both particular forms of mutualism. Interactions ➁ to ➃ are bidirectional.

Microbial interactions are crucial to the outcomes of bioprocesses. They are found in both natural communities and man-made co-cultures, where they are deliberately favored. The different types of interactions have been thoroughly detailed in some reviews (Großkopf and Soyer, 2014; García et al., 2019; Rodríguez Amor and Dal Bello, 2019). However, the terminology for the different types of positive interactions may slightly differ between authors. We therefore decided to specify what we consider to be positive interactions occurring between microorganisms. In all cases, positive interactions are defined as an improvement in the fitness of at least one partner involved in the interaction. Because interactions are often studied between two microorganisms, we will also present them in pairs in the sections below (Figure 2).

First, we should consider commensalism, which refers to an increase in the fitness of one partner in the interaction with no cost or benefit for the other. This is the only unidirectional positive interaction between two microorganisms. A notable example of this mode of interaction is that seen in Swiss-type cheese. Indeed, LAB strains contribute to the growth of PAB strains via two mechanisms: LAB produce lactic acid, which is further metabolized into propionic acid, acetic acid, and carbon dioxide by PAB (Smid and Lacroix, 2013) and they hydrolyze cheese proteins, thus supplying PAB with peptides and free amino acids (Gagnaire et al., 2001).

The other type of positive interaction is bidirectional. Three types of bidirectional positive interactions can be found in the literature, it can be difficult to distinguish them from each other: cooperation, mutualism and syntrophy, the most common being cooperation and mutualism. Cooperation implies the increased fitness of nearby cells that share a given genotype. This means that the interacting partners produce and use the same common goods, i.e., homotypic cooperation (Rodríguez Amor and Dal Bello, 2019). For instance, *Saccharomyces cerevisiae* cells are able to flocculate when they are challenged by environmental stress. This cooperative trait relies on the production of a cell-wall protein (FLO1) which enables all cells expressing FLO1 to adhere together (Smukalla et al., 2008). Unlike cooperation, mutualism refers to an increased fitness of both interacting partners that do not produce or use the same common goods, i.e., heterotypic cooperation (Rodríguez Amor and Dal Bello, 2019). Mutualism can be observed in traditional fermented food products such as kefir where yeasts supply essential amino acids and vitamins to LAB, which in turn lower the pH for yeasts (Cheirsilp et al., 2003; Stadie et al., 2013). Syntrophy is another mutual interaction that cannot be overcome by simply adding a co-substrate or any type of nutrient (Morris et al., 2013). In a co-culture of two microorganisms, syntrophy occurs when one strain benefits from the goods produced by the other strain, which in turn benefits from the consumption of these goods. The best known example is the H_2_-mediated syntrophic interaction between secondary degraders and methanogens (Schink, 1997).

Division of labor (DOL) benefits from special status in the literature: it is described in natural communities and reproduced in co-cultures. Also referred to as functional specialization, the DOL is an association of multiple strains in order to perform complex tasks. In a recent article, Giri et al. (2019) suggested four criteria to determine whether an interaction does indeed constitute a DOL. The first is functional complementarity, meaning that every partner in the interaction carries out a function more efficiently that the others. Second, the interaction needs to involve a synergistic advantage and thus needs to be bidirectional. The third criterion is negative frequency-dependent selection, meaning that the interaction can be sustained for a long period. Finally, a positive assortment is necessary, which will be favored by natural selection. For example, during the nitrification process, ammonia is first of all oxidized to nitrite by ammonia-oxidizing microorganisms (AOM) and then to nitrate by nitrite-oxidizing bacteria (NOB). This sequential substrate conversion maximizes ATP production and hence growth rates. The interaction between AOM and NOB is complementary, synergistic, ecologically stable, and displays signs of positive assortment, thus suggesting that nitrification fulfils all the criteria to classify as a DOL. A DOL is often artificially or synthetically reproduced in bioprocesses strategies, but in this context only the first two criteria need to be fulfilled to qualify the interaction as a DOL.

The dynamic aspects of microbial interactions are rarely mentioned when describing their different types. Over a long timescale, microbial communities display a relative stability while displaying an alternance of dominant species or even strains at a reduced timescale of several days. In this way, the interactions between microorganisms are not fixed in time and may vary depending on environmental conditions or the occurrence of disturbances. In co-cultures, stability is less of a focus because co-cultures are essentially achieved using batch cultures that only last for a few days or months. However, the population dynamic may also be important in the case of sequential interactions that can result in commensalism.

### Mechanisms Underlying Positive Interactions

#### General Mechanisms for Positive Microbial Interactions

**FIGURE 3:**
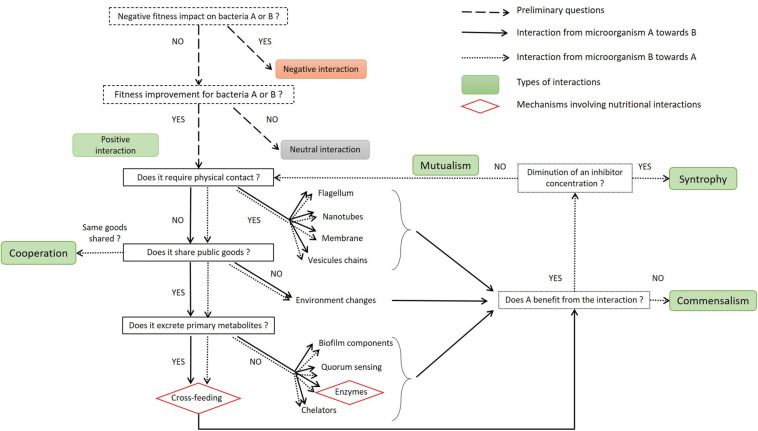
Decision tree to characterize the mechanism underlying positive interactions between two microorganisms. The dotted squares contain preliminary questions to establish whether this is a case of positive interaction or not, or supplementary questions to establish its type. In the case of bidirectional interactions, the decision tree can be read twice: the first reading aims to characterize the effect of microorganism A on B (plain arrows), and the second aims to characterize the effect of B on A (dotted arrows). The sequential questions are designed to establish whether the interaction:
–is direct or indirect,–engages a sharing of public goods or is due to physicochemical changes in the medium,–is reliant on secreted primary metabolites or not.

As the principal factor influencing process outcomes, microbial interactions need to be characterized accurately if they are to be properly applied (Li et al., 2013). The decision tree presented in Figure 3 illustrates how to describe these positive interactions. Positive interactions can require physical contact between the microorganisms. This contact may involve the flagellum, nanotubes, membrane, or vesicle chains. It is important to note that the molecules shared by direct contact are not detailed in Figure 3 but may include some of those shared through diffusion in the medium. The pool of molecules available in the medium is referred as public goods; they can be costly to produce and are equally available to all microorganisms in the medium (Cavaliere et al., 2017; Giri et al., 2019). A wide variety of molecules can be shared: siderophores, enzymes, biosurfactants, biofilm matrix components and QS molecules, as reviewed by West et al. (2007). If they are not used by the interacting partner, these molecules do not qualify as public goods but may modify the physicochemical properties of the medium so that they better suit growth conditions for the partner (i.e., CO_2_ production, increase or decrease of pH/redox). A distinction is made regarding the nature of the public goods thus produced: they may be nutritional or non-nutritional for the interacting partner; in other words, they can be used directly for growth or to improve its activity. Non-nutritional goods may include QS or biofilm components that can influence the spatial structure of the co-culture (Monnet et al., 2014). If nutritional goods such as amino acids or sugars are secreted, then the microorganisms interact via cross-feeding. Another nutritional interaction involves extracellular enzymes that hydrolyze proteins or complex polysaccharides directly in the medium.

#### The Interaction Gets Physical: The Case of Direct Transfers

As explained by Zengler and Palsson (2012), microbial interactions may require direct physical contact between interacting microorganisms. The best known way to create physical contact is to use a flagellum-mediated system which enables the transmission of a signal to a specific partner by recognizing cell-surface proteins (Shimoyama et al., 2009). In the case of the syntrophic interaction between methanogens and secondary degraders, a specific inter-species cell-to-cell recognition system is necessary to ensure efficient hydrogen transfer. Indeed, random cell-to-cell associations with other microbial species may cause a deterioration of methanogenic metabolism. The role of a flagellum is therefore not just motility but also includes adhesion and environmental sensing (Kouzuma et al., 2015).

The use of nanotubes has also been described as being necessary to establish positive interactions between microorganisms. Pande et al. (2015) showed that in a well-mixed environment, *E. coli* can connect with other bacterial cells via membrane-derived nanotubes and then use them to exchange cytoplasmic constituents.

The direct delivery of molecules can also be mediated by membrane-membrane contact. This was demonstrated by Benomar et al. (2015) regarding the formation of an artificial co-culture of *Clostridium acetobutylicum* and *Desulfovibrio vulgaris* in which a cell–cell interaction was associated with an exchange of molecules. This induced changes to metabolic pathways, and enabled the growth of *D. vulgaris* despite a shortage of nutrients. Remis et al. (2014) revealed the presence of a fourth direct communication system, showing that *Myxococcus xanthus* was capable of producing a network of outer membrane extensions in the form of vesicles and vesicle chains that can interconnect cells.

The requirement for physical contact between positively interacting microorganisms can be assessed using different methods. If interactions occur in sequential cultures (Ponomarova et al., 2017), using co-culture devices (Paul et al., 2013) or inactivated microorganisms (Cheirsilp et al., 2003), this means that no physical contact is required. However, identifying the nature of a physical contact is complicated because this requires the use of electron and fluorescence microscopy and identification of the flagellum, nanotubes or vesicle constituents.

#### Influence of Spatial Structure and Quorum Sensing

Spatiality plays a key role in microbial interactions, even those of an indirect type. Indeed, spatially structured media favor the stabilization of mutualistic cross-feeding (Pande et al., 2016). It is known that spatial assortment strengthens local interactions, prevents cheaters from taking advantage (MacLean and Gudelj, 2006) and improves resilience in the face of environmental stresses (Lee et al., 2014). Kim et al. (2008) revealed the importance of spatial structure to bacterial community interactions using a mathematical model to show how it influences or stabilizes both negative and positive interactions. One undeniable piece of evidence of the importance of spatial structure when considering bacterial interactions is biofilms. For more details in this field, the reader is referred to the interesting review by Nadell et al. (2016), which explain how positive or negative interactions (i.e., competition) are influenced by the spatial arrangement of different strains in biofilms.

The molecules involved in QS can modulate the spatiality between interacting microorganisms. In fact, two types of signaling molecules can modulate the expression of specific genes responsible for the synthesis of biofilm components, as well as bacteriocins, the conjugal transfer of plasmids, and a stress response (Di Cagno et al., 2007; Fontaine et al., 2007). These molecules are small signaling hormone-like autoinducers that accumulate and trigger cascade events when a quorum is reached (threshold concentration). The autoinducing peptide (AIP), also called peptide pheromone, is a species-specific communication signal found exclusively in Gram-positive bacteria. Autoinducer-2 (AI-2) furanones are universal signaling molecules that are also used in QS. QS manipulations are strategies that are sometimes implemented to increase yields in bioprocesses (Shong et al., 2012). It is in fact possible to modify synthesis of the signal molecule, sensitivity to the signal and the specificity of the response. Molecules involved in QS are important to bacterial dynamics in communities and co-cultures and can also affect the sensory quality and safety of foods as they may contribute to the elimination of undesirable microorganisms (spoilers or pathogens) and favor the development of those being targeted (Rul and Monnet, 2015).

In the case of unstructured media, the exchange of public goods, and particularly those of a nutritional type, may be trickier. In fact, this requires a subtle equilibrium between the metabolites produced, used and exchanged to obtain positive interactions between microorganisms (Bachmann et al., 2011). Interacting microorganisms need to produce more of the essential nutrients that they themselves require to sustain the growth of both partners, while avoiding cheating phenomena. In the case of proteolytic strains, a peptide concentration gradient surrounds the cells, so there is a specific location for nitrogen sources in the extracellular medium. This explains why proteolytic strains do not disappear in a co-culture that involves both proteolytic and non-proteolytic strains: the substrates are firstly used by the proteolytic cells and then diffused toward non-proteolytic cells (Bachmann et al., 2011).

#### Nutritional Dependencies Make Strong Allies

The sharing of primary metabolites is crucial in many positive interactions, whether they occur in natural communities, artificial or synthetic co-cultures. It is possible to distinguish two ways by which microorganisms supply these primary metabolites (Figure 3). The first is the sharing of hydrolytic extracellular enzymes such as invertases, lipases and proteases, which transform the substrates directly available in the public pool of molecules (Cavaliere et al., 2017). The second concerns cross-feeding, i.e., the phenomenon by which one microorganism (referred to as the donor) takes in a primary substrate and converts it into a product excreted as a public good, which is subsequently used by an interacting partner, referred to as the receiver. Cross-feeding has been extensively reviewed by D’Souza et al. (2018).

The evolution of natural communities has been described as the Black Queen Hypothesis, which implies that positive interactions are formed in complex habitats and strengthened through gene loss (Morris et al., 2012), thus creating dependencies between interacting microorganisms (Sachs and Hollowell, 2012). Over time, complex interactions develop between strains belonging to the same biotope, to the point where isolated strains can no longer sustain themselves. This theory highlights the fact that dependencies – particularly those of a nutritional type – between microorganisms not only improve their fitness but also strengthen their association against competitors, cheaters and environmental stress. Computational analyses have shown that metabolic dependencies are major drivers of species co-occurrence in nature (Zelezniak et al., 2015). Metabolism overflow is in fact frequent in microorganisms and can serve other community members (Paczia et al., 2012).

In artificial co-cultures, many positive interactions can be explained by extracellular enzyme sharing and cross-feeding that form the basis for the occurrence of positive interactions (Cavaliere et al., 2017), as exemplified below. Zuroff et al. (2013) used the cellulosic activity of *Clostridium phytofermentans* to supply fermentable simple carbohydrates to *Candida molischiana* to enable the production of ethanol. In milk fermentation, the sharing of extracellular protease, especially in LAB species such as *Lactococcus lactis*, has been shown to be paramount to ensuring microbial interactions in cheese and fermented milks (Smid and Lacroix, 2013). Nutritional interactions also occur as a result of cross-feeding. Ponomarova et al. (2017) showed that *S. cerevisiae* can sustain the growth of LAB strains by secreting essential amino acids and vitamins. The same observation was made regarding the fermentation of kefir water made from various fruit juices in which the growth of *Liquorilactobacillus hordei* and *Liquorilactobacillus nagelii* was supported by the release of amino acids and vitamin B6 by *Zygotorulaspora florentina* (Stadie et al., 2013). The cross-feeding of small carbohydrates from the hydrolysis of lactose or maltose can also occur between LAB and yeasts (Gobbetti, 1998; Ponomarova et al., 2017).

To create synthetic co-cultures, the most relevant interactions to be encouraged are commensalism and mutualism based on cross-feeding (Jagmann and Philipp, 2014). This means that co-cultures should be based on the excretion of nutritional compounds to favor positive interactions between microorganisms. Numerous examples have shown that this strategy is efficient in partitioning metabolic roles and engineering a DOL (Zhou et al., 2015; Ding et al., 2016; Johns et al., 2016). For example, Bernstein et al. (2012) genetically modified two strains of *E. coli* so that one converted glucose to acetate and the other, which was glucose-negative, used the metabolic by-product to increase total biomass production. In synthetic co-cultures, metabolic pathways have also been modified using specific mutations so that strains can only grow under strict cross-feeding. These pathways often involved amino acids, for which the strains are modified to become auxotrophs or overproducers (Wintermute and Silver, 2010; Mee et al., 2014). However, according to our definitions, it appears that the sharing of public goods, and particularly extracellular enzymes, is also used in synthetic co-cultures to engineer a DOL. Bayer et al. (2009) managed to modify a strain of *S. cerevisiae* so that it produced methyl halides with the support of a cellulolytic strain of *Actinotalea fermentans*. For the conversion of xylan to ethanol, Shin et al. (2010) modified an *E. coli* strain to produce xylanase, thus providing the necessary substrate for a second strain of *E. coli* that had been modified to convert xylo-oligosaccharides into ethanol.

Cross-feeding also includes co-factors. For example, heme and quinones have been reported to switch the metabolism of LAB from fermentation to respiration (Seth and Taga, 2014). Respiring LAB display faster growth rates, improved long-term survival and changes to their metabolism (Pedersen et al., 2012). Corrinoids, which notably include vitamin B12, are also involved in nutritional cross-feeding, as they are essential for bacterial growth. However, a high proportion of bacteria and almost 70% of LAB lack the ability to produce corrinoids *de novo*. This means that bacteria need to procure them from their environment, either directly via the medium or through the activity of other microorganisms (Seth and Taga, 2014).

## Lab Co-cultures to Multiply Functionalities

Although co-cultures have proved their worth in numerous applications, LAB-LAB co-cultures seem to have been somewhat neglected. In fact, yogurt is the only example of a LAB co-culture that is very widely exploited and whose interactions have been well characterized. LAB are endowed with a broad range of functions that could be multiplied with artificial co-cultures, particularly in food applications. Further, LAB have particular metabolic traits that favor the artificial establishment of nutritional dependencies.

### LAB: Functional Microorganisms in Food Applications

LAB are Gram-positive, non-sporulating, facultative anaerobic and acid-tolerant bacteria. LAB species can be homofermentative, meaning that they mainly produce lactic acid; or heterofermentative, meaning that they also produce acetic acid, ethanol, CO_2_ and formic acid (Wright and Axelsson, 2019). They are found in a variety of nutrient-rich food environments, and especially in dairy products, vegetables, cereals and meat, as autochthonous flora or added starter cultures. They also form part of the natural microbiota of animal hosts, where they are found in the gastrointestinal tract, the urogenital tract, oral cavity and skin. LAB are used as functional cultures because they contribute to the final organoleptic, nutritional, health and sanitary properties of food products. Zheng et al. (2020) suggested a new classification for the large group that represent LAB. They are now divided into five families: *Aerococcaceae*, *Carnobacteriaceae*, *Enterococcaceae*, *Streptococcaceae*, gathering 7, 16, 7, and 3 genera, respectively, and *Lactobacillaceae* now gathering 31 genera, while the former *Lactobacillus* genus has been expanded to 25 different genera. The most frequently isolated LAB species belong to the genera *Lactococcus*, *Lactobacillus*, *Lactiplantibacillus*, *Streptoccocus*, *Oenococcus*, *Leuconostoc*, and *Enterococcus*. New LAB species are constantly being described, and the total number of LAB species is evaluated at more than 200 (Holzapfel and Wood, 2014).

#### Organoleptic Changes Induced by LAB

LAB play an important role in organoleptic changes to food products. They actively contribute to both flavor and texture changes in fermented foods, and particularly dairy products. For example, in fermented dairy products, the proteolytic activity of LAB strains is crucial to both transformations. Indeed, the sapid peptides and amino acids produced by LAB participate in overall flavor perception and free amino acids are aroma precursors. LAB also produce different aroma compounds such as diacetyl and acetaldehyde, which are easily identifiable in the overall aroma note of yogurt (Zourari et al., 1992; Routray and Mishra, 2011), and a wide variety of aroma compounds derived from amino acid catabolism (Smid and Kleerebezem, 2014).

LAB activity is also essential to textural properties. Like many other bacteria, they can produce several types of polysaccharides or glycans that are natural biopolymers of carbohydrates and display enormous structural diversity (Ruas-Madiedo et al., 2002; Bernal and Llamas, 2012). Bacterial exopolysaccharides (EPSs) are either loosely bound to the cell surface or released into the surrounding environment, and they can change the texture of food products (Rahbar Saadat et al., 2019), whether through the production of peptides or their proteolytic activity (Lacou et al., 2016).

#### Nutritional Quality Improvements Induced by LAB

LAB also enhance the nutritional quality of foods; they may either improve nutritional intake and digestibility or reduce the presence of anti-nutritional compounds. LAB can produce vitamins (LeBlanc et al., 2011); for example, *Leuconostoc mesenteroides* and *Latilactobacillus sakei* produce riboflavin and folic acid in kimchi (Jung et al., 2011) and *L. lactis* produces K2 vitamin (menaquinones) (Morishita et al., 1999).

The EFSA recognizes that the LAB species found in yogurt are able to alleviate the symptoms of lactose malabsorption (Efsa Panel on Dietetic Products Nutrition and Allergies, 2010). This condition is due to a lack of human lactase in the small intestine. LAB diminish the lactose content during fermentation and contain the β-D-galactosidase enzyme that may further be active in the gastrointestinal tract (Shah, 2015). They may also reduce intestinal discomfort due to the non-digestible oligosaccharides present in many plants (such as stachyose and raffinose in legumes) by hydrolyzing these compounds in fermented plant-derived products. Improved protein digestibility has also been observed *in vitro* in sourdough (Bartkiene et al., 2012). The proteolytic activity of LAB may also decrease the protein allergenicity of food products such as soybean (Song et al., 2008).

Finally, LAB can also reduce the quantities of anti-nutritional compounds such as phytates that are present in some plant-based products (García-Mantrana et al., 2015), either by lowering the pH of the medium, which reactivates endogenous plant phytase(s), and/or by producing bacterial phytase(s) that can release the inositol moieties of phytates.

#### Health Benefits

LAB exert beneficial effects on hosts as they can produce bioactive molecules, either in fermented products or directly in the gastrointestinal tract. According to the FAO and WHO, probiotics are “live microorganisms which, when administered in adequate amounts, confer a health benefit to the host”. The most commonly used probiotic bacteria belong to LAB, and especially those in the former genera *Lactobacillus* and *Enterococcus* (Tamang et al., 2016a). Bioactive peptide production from LAB is often related to health benefits. Bioactive peptides are released from proteins by microbial or non-microbial enzymatic hydrolysis (Fitzgerald and Meisel, 2003). Because of their proteolytic system, LAB fermentation process can release these peptides in a protein-based medium (Venegas-Ortega et al., 2019). Bioactive peptides can act as immune modulators (Qian et al., 2011), and are antihypertensive by inhibiting angiotensin-converting enzyme (ACE) (Phelan et al., 2014), as in yogurts (Papadimitriou et al., 2007) and antioxidants as in sourdough (Coda et al., 2012) and yogurt (Sabeena Farvin et al., 2010). Antioxidant peptides have been found in soybean milk fermented by LAB and these can effectively eliminate free radicals (Liu et al., 2017). Antioxidant properties may also be the consequence of EPS production (Rahbar Saadat et al., 2019).

Other health benefits are related to the consumption of products fermented using probiotic LAB strains. Indeed, yogurt consumption produced some interesting results versus type 2 diabetes in a meta-analysis of dairy intake (Gijsbers et al., 2016). LAB are also involved in anti-cancer activities. For example, in milk, *Lactobacillus acidophilus* produces conjugated linoleic acid, an anti-carcinogenic agent (Macouzet et al., 2009). LAB also produce antimicrobials compounds whose properties can also contribute to the establishment of probiotic strains in the host (Kommineni et al., 2016) and thus counteract pathogenic bacteria in the gastrointestinal tract.

#### Food Preservation and the Safety of LAB

Another function of LAB is food preservation, generally considered to be the principal reason why they have been used empirically for centuries in traditional fermented foods. LAB inhibit the growth of numerous pathogens and spoilage microorganisms, by both lowering the pH as a result of lactic acid production and by producing a wide variety of antimicrobial compounds. Lactic acid induces ionic disruption (Warnecke and Gill, 2005), changes to the fatty acid composition of cell membranes (Cotter and Hill, 2003), and changes in transcriptional responses (Kirkpatrick et al., 2001) or oxidative stress within *Bacillus cereus* cells (Mols and Abee, 2011). In the presence of oxygen, some LAB are able to produce hydrogen peroxide which causes DNA damage, protein oxidation and membrane disruption in *Pseudomonas*. The ethanol produced by some LAB species, in association with other molecules, induces damage to cell membranes and denatures proteins (Ross et al., 2002). Diacetyl, involved in the butter-like aroma expected in some fermented dairy products, inhibits some Gram-negative bacteria (Kang and Fung, 1999). The overall antimicrobial impact of LAB therefore lies in the synergistic effect of a range of metabolites they produce. Organic acids from LAB also inhibit pathogenic and spoilage fungi (Crowley et al., 2013). The ability of LAB to inhibit microorganisms is supplemented by the production of bacteriocins, which are multifunctional peptides produced in the ribosome and display antimicrobial activity at particular concentrations (Chikindas et al., 2018). Bacteriocins are clustered in different classes depending on their structure, genetics and mode of action. They have been shown to inhibit a wide range of food spoilage bacteria and fungi (Zacharof and Lovitt, 2012; Leyva Salas et al., 2017). The addition of bacteriocins to food can indeed lower pathogen levels in a variety of products (O’Sullivan et al., 2002). Nisin, a bacteriocin produced by *L. lactis*, is the most widely exploited bacteriocin. It creates pores in the bacterial membrane that create leakage in Gram-positive bacteria (Bierbaum and Sahl, 2009). The use of LAB producing antibacterial and antifungal agents is a natural alternative to the addition of chemical preservatives. They can also improve the flavor of certain fermented foods (Younes et al., 2017).

Despite the wide and documented use of LAB in food production, safety concerns also need to be considered when they are added deliberately to foods. Two statuses are used to qualify LAB safety. A list of 48 LAB species benefit from a Qualified Presumption of Safety (QPS) status defined by the European Food Safety Authority (EFSA). *Enterococcus spp.*, known for its ability to develop antibiotic resistance, is not included on the QPS list. However, it is important to note that some other species currently used as starters in foods do not (as yet) figure on the QPS list. QPS status offers species a safety qualification but does not preclude an evaluation of the risk involved in any (LAB) strain before it is used as a starter. There are two main risks, which depend on the LAB species or strains employed: the transfer of determinants of antibiotic resistance and the production of toxic or deleterious compounds such as biogenic amines and D-lactic acid. The selection of strains for use in foods must therefore be very thorough. The second status, Generally Regarded As Safe (GRAS) and determined by the United States Food and Drug Administration (FDA) qualifies LAB at the strain level.

### Promoting LAB Positive Interactions in Co-cultures

#### Quorum Sensing and Its Technological Implications

QS is probably the most widely studied bacterial communication system. It regulates bacterial gene expression as a function of cell density and environmental conditions. It offers an adaptive advantage to specific bacterial populations in complex communities as well as to those which are enriched, such as in traditional fermented foods. In that regard, QS plays an important role in many of the microbial successions that occur during food fermentation: it can enhance the growth of specific species or strains while inhibiting others (Johansen and Jespersen, 2017). However, any evidence of positive interactions mediated by AIP between LAB in co-cultures is rare or even contrary. For example, it was observed that the growth and viability of *Lactiplantibacillus plantarum* DC400 was enhanced when cultured with *Fructilactobacillus sanfranciscensis* and *Furfurilactobacillus rossiae* in sourdough, when QS-related genes responsible for stress response were activated (Di Cagno et al., 2009). Also, the growth and survival of the AIP-producing starter *L. plantarum* from Spanish-style olives increased when co-cultivated with AIP-inducing *Enterococcus faecium* and *Pediococcus pentosaceus* strains (Ruiz-Barba et al., 2010). In contrast, the survival of *L. plantarum* was not enhanced when co-cultivated with an AIP-inducing *L. lactis* strain (Caballero-Guerrero et al., 2013). Further, AIP production was reduced in both *L. plantarum* and *E. faecium* isolated from fermented vegetables during co-cultivation (Domínguez-Manzano and Jiménez-Díaz, 2013).

Although QS systems offer an important colonization advantage to LAB in complex communities, evidence of positive interactions in LAB co-cultures has yet to be produced. It was recently shown that exogenous synthetic AI-2 exerted positive effects on the growth of *E. faecium* and *Limosilactobacillus fermentum*, and on the cell density of *E. faecium* under acidic and alkaline stresses (Gu et al., 2020). Therefore, associating AI-2 producing LAB strains might lead to positive interactions. However, QS requires a certain cell density to regulate gene expression, thus implying that at least one partner in the interaction is able to grow independently. In addition, this phenomenon is only observed on solid or semi-solid structured matrices.

Nevertheless, QS is of considerable interest in food technologies as it can enhance food safety and quality through the production of antimicrobial compounds (Ruiz-Barba et al., 2010; Kareb and Aïder, 2020). In the near future, QS could be used to control microbial behavior and thus improve the quality of foods and beverages, for example by favoring the growth of LAB that enhance flavor and texture or by inhibiting the growth of spoilage or pathogenic microorganisms. QS also has a significant role to play in the probiotic functionalities of LAB strains, such as resistance to harsh environments; e.g., gastric acidity (Johansen and Jespersen, 2017).

#### Manufacturing Nutritional Dependencies Between LAB

LAB are found in in the majority of naturally fermented products ranging from milk to meat, as well as in various plants, which mostly offer nutrient-rich media (Tamang et al., 2016b). Their widespread occurrence can be explained by the fact that LAB display a huge diversity of phenotypes that vary within and between species. LAB can utilize many different substrates, notably carbohydrates and proteins (Duar et al., 2017). Some LAB species have also adapted insofar as they have developed auxotrophy for many nutrients (Teusink and Molenaar, 2017). These two characteristics make them ideal partners to create LAB-LAB positive interactions if account is taken of the composition of the medium, the possibility that one or other of the partners involved in the interactions can supply essential nutrients and by limiting potential substrate competition.

The auxotrophies present in LAB are markedly dependent on the biotopes from which they originate. They are therefore species- and strain- dependent. Many LAB strains have been shown to lack the capacity to produce the precursors of RNA and DNA: they are auxotrophic for nucleosides and nucleic acids (Kilstrup et al., 2005). LAB also grow poorly or even not at all in environments where vitamins (particularly from the B group), peptides and amino acids are not available. The development of chemically defined media (CDM) has offered crucial clues to identifying the nutritional requirements of LAB strains. For example, *L. lactis* strains are auxotrophic for 14 of 20 amino acids (Cocaign-Bousquet et al., 1995), *L. plantarum* and *L. mesenteroides* require between 3 and 11 amino acids (Teusink et al., 2005; Kim, 2012), while *Lactobacillus johnsonii* is unable to synthetize any of them (Hammes and Hertel, 2015). Requirements in leucine, isoleucine, valine, methionine, and glutamate are the most common. Most LAB strains also need exogenous sources of vitamins for growth, mainly pantothenate, pyridoxine, riboflavin, niacin and biotin (Zhang et al., 2009; Wegkamp et al., 2010; Aller et al., 2014), whereas some LAB strains are able to produce vitamins (LeBlanc et al., 2011; Capozzi et al., 2012). Finally, LAB are also known to be heme-auxotrophic (Gruss et al., 2012).

LAB also differ in their ability to utilize external resources. LAB can procure amino acids from their environment by using the peptide intracellular transport system. They can import peptides via an oligopeptide transport system (Opp) as well as di- and tri-peptides (DtpT and Dpp). Intracellular peptidases then hydrolyze these peptides into assimilable amino acids. In addition, some LAB strains possess a cell-envelope proteinase (CEP) that hydrolyzes the proteins present in the medium (Kunji et al., 1996). Five different types of such enzymes have been identified in LAB, including PrtP in *L. lactis* and homologs to PrtP in *Lacticaseibacillus paracasei*, PrtH in *Lactobacillus helveticus*, PrtR in *Lacticaseibacillus rhamnosus*, PrtS in *S. thermophilus*, and PrtB in *L. delbrueckii* subsp. *bulgaricus* (Savijoki et al., 2006). Depending on the LAB strain, the proteolytic equipment may differ in terms of the nature of peptidases and transport systems. In some species, the presence of CEP (Liu et al., 2010) and their number differ depending on the strain: *L. helveticus* strains can contain up to four different CEPs, in contrast with other LAB species (Sadat-Mekmene et al., 2011). LAB also display considerable differences in their abilities to ferment carbohydrates. While glucose is commonly preferred by many LAB strains, they present disparities in the use of other mono- and oligo-saccharides (Hayek and Ibrahim, 2013). Sugar intake depends on permeases, ATP-driven transporters or phosphoenolpyruvate: sugar phosphotransferase systems (PEP:PTS) present in the LAB genome (Poolman, 1993). For example, lactose intake is mediated by a PEP:PTS system in *L. lactis* whereas it is mediated by a permease in *L. delbrueckii* subsp. *bulgaricus* (Leong-Morgenthaler et al., 1991). Oligosaccharide intake requires initial enzymatic cleavage (with β-galactosidase, α-galactosidase or sucrase) in order to form monosaccharides which can enter the functional fermentation pathways. Sugars that are not metabolized are excreted into the medium (Wright and Axelsson, 2019). This is the case for *S. thermophilus*, which excretes the galactose moiety of lactose, which is further used via the Leloir pathway by *L. bulgaricus* in yogurt, for example (Zourari et al., 1992).

Nutritional dependencies are the key to creating artificial LAB co-cultures of metabolically dependent partners that positively interact through different mechanisms: the cross-feeding of vitamins or sugars or the sharing of public goods such as the CEP, which supplies peptides and free amino acids. A study by Settachaimongkon et al. (2014) investigated the interactions between proteolytic and non-proteolytic strains of *S. thermophilus* in co-culture with *L. delbrueckii* subsp. *bulgaricus* and showed that dependency between two LAB strains is essential for the interactions to be successful. The mutualistic interaction observed in yogurt does not occur when *S. thermophilus* has its own CEP. Another strategy might be to use strains which can hydrolyze complex sugars in order to furnish simple sugars to a LAB strain that does not possess the necessary enzymes. The fact that LAB are able to utilize a rich diversity of carbon sources for energy is an advantage as it limits resource overlaps and hence competition.

## Perspectives for Lab – Lab Co-cultures in Food Fermentation

In this review we have shown that co-cultures can be used for substrate conversion, multiplication of the functional traits expressed and the optimization of bioprocesses. We have also showed that the stronger the microbial interactions, the greater are the outputs. The positive combination of microbial strains seems mainly due to metabolic dependencies between the interacting partners. Our aim was to demonstrate that this is particularly relevant for the co-culture of LAB, which both (1) offers a broad range of functionalities to food products, such as organoleptic, sanitary, nutritional and health properties, and (2) displays a high level of metabolic diversity that enables the construction of nutritional dependencies between strains and thus the creation of positive interactions within co-cultures.

Artificial LAB co-cultures could be used to ferment new matrices containing different resource types that have more or less been exploited until now. In a context of developing more sustainable and healthy food products, animal-sourced proteins need to be consumed less, with a greater preference for plant-based proteins because of environmental impacts and the increasing need for proteins in general. It is therefore relevant to look for new ways to valorize plant resources, and particularly plants with a high protein content. Fermentation by LAB has already proved crucial in various traditional plant-derived products (Tamang et al., 2016b). By means of appropriate strain selection and associations to promote positive interactions, LAB could therefore make an important contribution to alleviating unpleasant sensory characteristics as well as nutritional and digestive disadvantages. They can in fact modify aroma profiles, and lower the concentrations of phytates and oligosaccharides responsible for intestinal discomfort. Kimoto-Nira et al. (2012) introduced the idea that artificial LAB co-cultures could also be used to ferment mixes of milk and plant-based substrates. They created a co-culture of *L. lactis* and *L. raffinolactis* that better acidified milk when the two strains grew together. The non-proteolytic *L. raffinolactis* strain was able to degrade melibiose, raffinose and stachyose which cause intestinal discomfort, while *L. lactic* was used for its production of a desirable flavor. It is important to note that when describing the interactions from an “end-product” point of view, positive interactions can involve either positive microbial interactions or no interactions. In fact, in a medium where both LAB grow without influencing one another, the product will gather more functions than if produced in a monoculture. It might therefore be possible to use a “pilot” strain responsible for the basic fermentation process (i.e., carbohydrate utilization) and complementary strains to add functionalities to the final product (i.e., production of aroma compounds or bioactive peptides).

Ultimately, a clearer understanding of positive interactions between LAB strains may lead to more efficient starters, as well as improvements to their production. Indeed, the mechanisms underlying interactions offer insights into possible LAB associations that might be more appropriate, depending on the results targeted. It may also enable the improved control of fermentation processes, leading to improved reproducibility. Further, starters are usually produced separately because of the disturbing effects on microbial viability of concentration, freeze-drying and storage steps. It is, however, possible to imagine co-cultures of LAB strains that would enable higher survival rates during these stressful processes, notably through an increased production of EPS, known to be a cryoprotectant. Finally, a clearer understanding of LAB interactions and their mechanisms could also facilitate the preservation of complex microbial assemblies, such as those found in cheese, and the cultivability of strains from complex environments.

The production of extracellular vesicles (EVs) (also referred as membrane vesicles) by LAB is most definitely a vector for interactions that should be considered in future studies. EVs are lipid bilayer-enclosed spherical structures which range in size from 20 to 300 nm and are released by cells from all living kingdoms (Kim et al., 2015; van Niel et al., 2018; Gill et al., 2019). They play a pivotal role in cell-to-cell communication through their ability to transport bioactive molecules (proteins, nucleic acids, lipids, metabolites, signaling molecules) from donor to recipient cells. In Gram-positive bacteria, most studies have been conducted on EVs generated by pathogenic bacteria (Brown et al., 2015; Liu et al., 2018b). They are implicated notably in the delivery of virulence factors, host cell modulation, antibiotic resistance, survival and microbial competition and cooperation. In LAB, EV production has been demonstrated in *L. plantarum*, *Enterococcus faecalis, L. rhamnosus*, *Limosilactobacillus reuteri*, and *Lacticaseibacillus casei* (Behzadi et al., 2017; Domínguez Rubio et al., 2017; Grande et al., 2017; Li et al., 2017) and to the best of our knowledge, their biological role was mainly considered with respect to LAB-host interactions. Considering the cargo molecules that Gram-negative and Gram-positive bacterial EVs can carry, as well as their ecological functions, LAB-derived EVs could also play crucial roles in microbial interactions, whether they are positive or negative. Indeed, they may be involved in nutritional interactions through the cross-feeding of nutritional compounds, and in the delivery of antimicrobial compounds (Liu et al., 2018a). The participation of EVs generated by LAB in the structure and function of microbial communities/co-cultures is a promising research area of considerable interest to the field of food biotechnology that still needs to be explored.

The co-culture of LAB offers new opportunities for food fermentation in terms of controllable outputs, product functionalization and resource utilization. The construction of such artificial co-cultures needs to be well-reasoned as their outcomes depend on numerous criteria: the targeted functionalities of the final product or end-product, the composition of the medium, abiotic conditions, the metabolic complementary of strains and of course, their safety. There is therefore a crucial need for a dedicated database gathering information on LAB genomes, known interaction networks, phenotypic screening results and isolated biotopes. Existing databases such as FoodMicrobionet^1^ can already provide valuable data and insights on the composition and relative abundances of the microorganisms in a food sample at a specific time (Parente et al., 2016), and Florilege^2^ gathers knowledge on microorganism biotopes and phenotypes from the literature using text mining tools (Falentin et al., 2017). These databases could be further exploited, supplemented and hopefully become interoperable in the future.

With a clearer understanding of LAB interactions, the development of databases, the use of mathematical modeling and meta-omics approaches, it may be possible to develop more sophisticated LAB co-cultures. Indeed, this review focuses on the example of a two-strain co-culture as a starting point and we believe that increased bacterial diversity will only offer more benefits to fermented food products and strengthen the bonds between associated strains.

## Author Contributions

FC collected the literature, wrote the manuscript, and the figures with the extensive contribution of VG and AT, who reviewed and edited each version. TN and EG reviewed the manuscript and the figures. All authors read and approved the final version of the manuscript.

## Conflict of Interest

The authors declare that the research was conducted in the absence of any commercial or financial relationships that could be construed as a potential conflict of interest.

## Footnotes

1 http://www.foodmicrobionet.org

2 http://migale.jouy.inra.fr/Florilege/#&welcome
